# The Intersection of the Staphylococcus aureus Rex and SrrAB Regulons: an Example of Metabolic Evolution That Maximizes Resistance to Immune Radicals

**DOI:** 10.1128/mBio.02188-21

**Published:** 2021-11-16

**Authors:** Aidan Dmitriev, Xingru Chen, Elyse Paluscio, Amelia C. Stephens, Srijon K. Banerjee, Nicholas P. Vitko, Anthony R. Richardson

**Affiliations:** a Department of Microbiology and Molecular Genetics, University of Pittsburgh, Pittsburgh, Pennsylvania, USA; b Department of Microbiology and Immunology, University of North Carolina at Chapel Hill, Chapel Hill, North Carolina, USA; New York University School of Medicine

**Keywords:** *Staphylococcus aureus*, coagulase-negative staphylococci, fermentation, immune radicals, metabolic evolution, metabolism, nitric oxide, redox signaling

## Abstract

Staphylococcus aureus is the most pathogenic member of the *Staphylococcaceae.* While it acquired an arsenal of canonical virulence determinants that mediate pathogenicity, it has also metabolically adapted to thrive at sites of inflammation. Notably, it has evolved to grow in the presence of nitric oxide (NO·). To this end, we note that the Rex regulon, composed of genes encoding dehydrogenases, metabolite transporters, and regulators, is much larger in S. aureus than other Staphylococcus species. Here, we demonstrate that this expanded Rex regulon is necessary and sufficient for NO· resistance. Preventing its expression results in NO· sensitivity, and the closely related species, Staphylococcus simiae, also possesses an expanded Rex regulon and exhibits NO· resistance. We hypothesize that the expanded Rex regulon initially evolved to provide efficient anaerobic metabolism but that S. aureus has co-opted this feature to thrive at sites of inflammation where respiration is limited. One distinguishing feature of the Rex regulon in S. aureus is that it contains the *srrAB* two-component system. Here, we show that Rex blocks the ability of SrrA to auto-induce the operon, thereby preventing maximal SrrAB expression. This results in NO·-responsive *srrAB* expression in S. aureus but not in other staphylococci. Consequently, higher expression of cytochromes and NO· detoxification are also observed in S. aureus alone, allowing for continued respiration at NO· concentrations beyond that of S. simiae. We therefore contend that the intersection of the Rex and SrrAB regulons represents an evolutionary event that allowed S. aureus to metabolically adapt to host inflammatory radicals during infection.

## INTRODUCTION

Staphylococcus aureus is the most pathogenic member of the genus Staphylococcus, which consists of numerous species associated with the colonization of skin, hair, fur, feathers, scales, and digestive tracts of virtually every terrestrial animal. While S. aureus is most commonly found to asymptomatically colonize the nares and skin of humans, it is often associated with localized skin and soft tissue infections (SSTIs) that can progress to more serious disease presentations, including sepsis, osteomyelitis, and endocarditis (1). Though some coagulase-negative staphylococci (CoNS) can possess virulence potential (e.g., S. epidermidis, S. lugdunensis, S. saprophyticus, and S. haemolyticus), none impact human health to the extent of S. aureus. Historically, S. epidermidis has been considered to be the closest relative to S. aureus. However, in 2005, S. simiae was first described after isolation from the feces of a South American squirrel monkey and was then deemed the closest relative to S. aureus (2). Simultaneously, divergent CC75 isolates from patients in Australia were dubbed nonpigmented S. aureus but eventually became recognized as a very closely related species, S. argenteus (3). More recently, another species isolated from nonhuman primates and bats in Africa has been described as S. schweitzeri (4). Now, it is generally accepted that S. aureus, S. argenteus, and S. schweitzeri comprise the S. aureus complex (SAC) and that S. simiae is the closest relative to the SAC, followed by S. epidermidis. Unlike members of the SAC, S. simiae is largely devoid of virulence factors and drug-resistant determinants (5). It does encode protein A, aureolysin, fibronectin binding proteins, clumping factors, and delta-toxin. However, it lacks alpha-, beta-, and gamma-toxins; serine/cysteine proteases; bi-component leukocidins; pigment; phenol-soluble modulins; and the type-7 secretion system, as well as the Isd iron acquisition system (5).

In the staphylococcal species with virulence potential, it has become appreciated that global metabolic regulators intersect with the expression of pathogenic traits. For instance, in S. aureus, the carbon catabolite protein CcpA is known to positively influence the expression of the virulence-coordinating Agr quorum-sensing system (6). Agr is also negatively impacted by the branched-chain amino acid-sensing CodY regulator (7). Some additional metabolic regulators may impact pathogenicity independently of Agr. These include SrrAB, which senses respiratory flux and has been shown to bind to the promoter and repress the superantigen toxic shock toxin, TSST-1, as well as Agr promoters, thereby indirectly influencing virulence factor production (8). Likewise, the cellular redox sensing Rex regulator binds directly to the bicomponent leukocidin, LukAB (9). SrrAB and Rex are also the most influential regulators that coordinate the response of S. aureus to host nitric oxide (NO·) (10). S. aureus is highly resistant to this immune radical, a trait that distinguishes it from CoNS (11). Rex contributes to NO· resistance by sensing the buildup of NADH due to the inhibition of cellular respiration by NO·. NADH binds to the C termini of S. aureus Rex dimer with nanomolar affinity and locks the N-terminal winged helix DNA binding motif in an inactive state (12). This releases Rex repression of a number of dehydrogenases that can oxidize NADH to NAD^+^, thereby reestablishing redox balance. In addition to contributing to S. aureus NO· resistance, Rex homologues are known to influence toxin production in Clostridium difficile and Bacillus cereus, survival of Streptococcus suis in macrophages, and biofilm formation in Streptococcus mutans (13–15). Thus, in several Gram-positive pathogens, Rex not only controls redox balance of the cell but also virulence trait expression.

The SrrAB two-component system senses decreased respiratory flux, presumably by surveying the level of reduced menaquinone analogous to the ArcAB system in Escherichia coli, though no direct evidence of this has been reported. However, it has indirectly been shown that the SrrAB regulon is active in a Δ*hemB* mutant (featuring a completely reduced menaquinone pool) but not in a Δ*menD* mutant (which lacks menaquinone altogether), even though neither mutant can respire (16). Furthermore, menaquinone analogues are inhibitory to S. aureus in an SrrAB-dependent fashion (17). Finally, like in the E. coli ArcAB system, there are redox-active cysteine residues in SrrB that form disulfide bonds *in vivo* and are required for full SrrAB activity (18). However, these residues are not conserved in all SrrAB orthologues in that they are absent in almost all staphylococcal species outside the SAC. Thus, another mode of sensing respiratory flux and/or the oxidation state of the menaquinone pool must exist for the majority of the SrrAB orthologs to function. When stimulated, SrrA drives the expression of both S. aureus cytochromes (cytochromes *aa*_3_ and *bd*), the anaerobic ribonucleotide reductase, pyruvate-formate lyase, NO·-detoxifying flavohemoprotein, as well as heme synthesis and iron-sulfur cluster repair proteins (10). Essentially, when respiratory flux wanes, SrrA increases the capacity of the electron transport chain to optimize the energy state of the cell. This is particularly important for NO· resistance since NO· detoxification, iron-sulfur (Fe-S) cluster repair, and maximization of cytochrome content all enable S. aureus to maintain positive energy balance in the presence of this immune radical (19).

Here, we show that the Rex regulon is significantly expanded in S. aureus compared with most other CoNS, save S. simiae and other members of the SAC. We show that this expansion is necessary and sufficient for NO· resistance and that this trait is not exclusively associated with S. aureus. We further show that SrrAB is autoregulated and Rex repressed, and therefore, NO· responsive, only in S. aureus. Thus, the merging of two metabolic regulons may represent an evolutionary event aimed at allowing S. aureus to achieve a metabolic state compatible with host inflammation.

## RESULTS

### The expanded Rex regulon is necessary and sufficient for NO· resistance.

**FIG 1:**
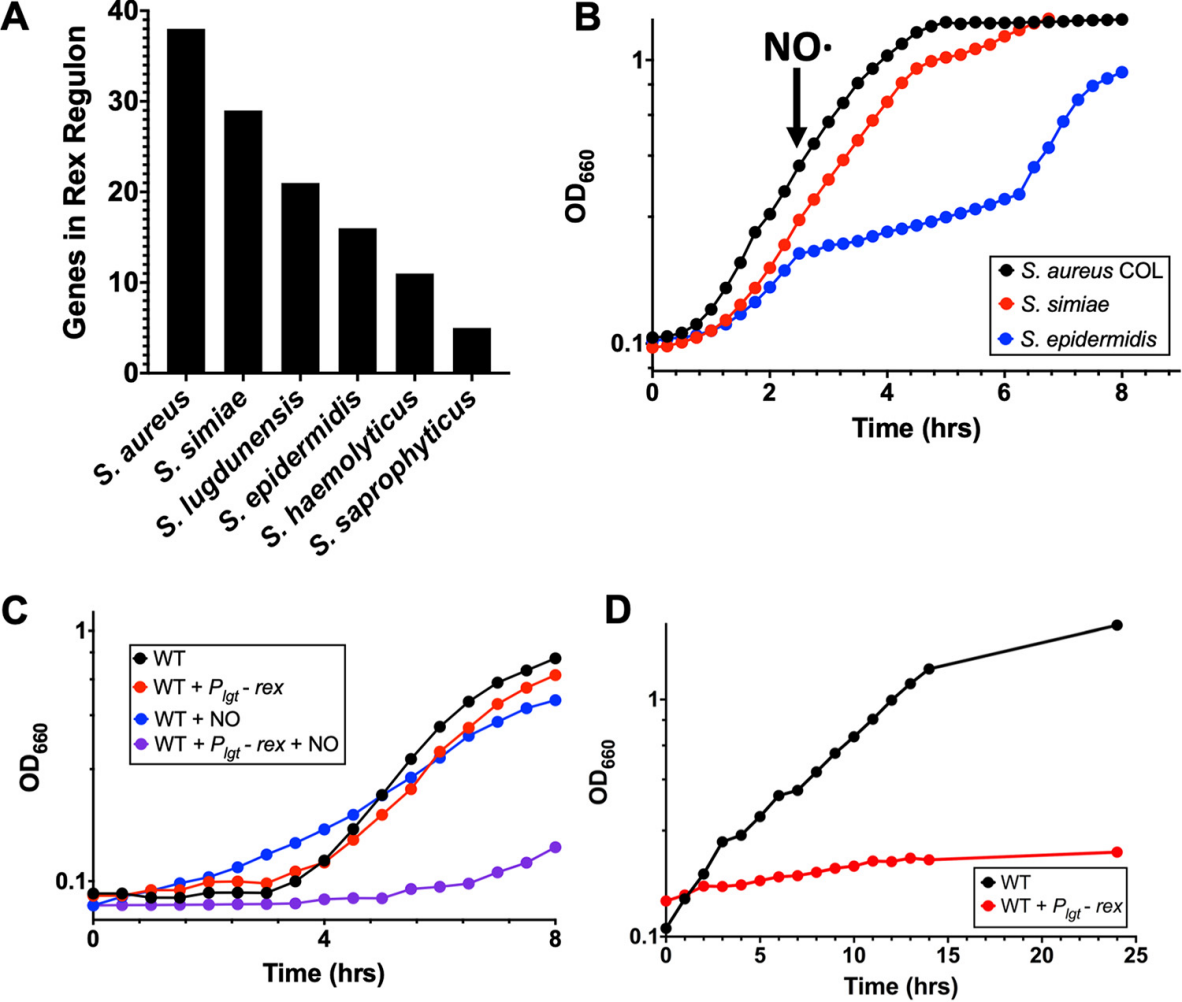
An expanded rex regulon is necessary and sufficient for staphylococcal NO· resistance. (A) Number of putative Rex-regulated genes from closely related staphylococcal species as determined by the presence or absence of a Rex binding sites in the promoter regions. (B) Representative growth curve of three replicates demonstrating that both S. aureus and S. simiae exhibit NO· resistance compared with S. epidermidis that lacks the expanded Rex regulon. NO· was administered as a mixture of 10 mM NOC and 12:1 mM DETA-NO. (C) Overexpression of Rex from the *lgt* promoter limits S. aureus growth in the presence but not the absence of NO·. Representative growth curve of three independent replicates using 10 mM DETA-NO as the NO· donor. (D) Overexpression of Rex from the *lgt* promoter limits growth anaerobically.

Inhibition of respiration in S. aureus, either by oxygen depletion or NO· exposure, is known to induce the expression of genes normally repressed by Rex. Given that S. aureus is highly resistant to NO· while other staphylococci generally are not, we sought to investigate the relationship between the Rex regulon and S. aureus NO· resistance. We conducted full-genome searches for Rex binding sites (TTGTGAW_6_TCACAA) located within 400 bp upstream of an annotated start codon and allowing a maximum of two mismatches in the following genomes: S. aureus COL, S. simiae CCM 7213, S. epidermidis RP62A, S. haemolyticus JCSC1435, S. saprophyticus ATCC 15305, Staphylococcus carnosus TM300, Staphylococcus pseudintermedius HKU10-03, S. lugdunensis HKU09-01, Staphylococcus warneri SG1, Staphylococcus pasteuri SP1, and Macrococcus caseolyticus JCSC5402 (Table S1 in the supplemental material). S. aureus possessed, by far, the most (38 putative Rex-regulated genes), followed by S. simiae with 29 putative Rex-regulated genes (Fig. 1A). NO·-sensitive S. epidermidis only encodes 16 putative Rex-regulated genes, and S. haemolyticus and S. saprophyticus encode even fewer (Fig. 1A).

10.1128/mBio.02188-21.6 TABLE S1 Survey of Rex regulons in related staphylococcal species. Orange column lists the distance that an identified Rex binding site is upstream from the start codon. The electrophoretic mobility shift assay (EMSA) column refers to reference 9 in which each identified promoter was subject to EMSA analyses. Each subsequent column lists the gene IDs for orthologs to genes in the S. aureus Rex regulon. Gray means the gene is absent, green means the gene is present and associated with a putative Rex binding site, and red means the gene is present but not associated with a putative Rex binding site. Darker-shaded boxes indicate the first gene in an operon, and lighter-shaded boxes indicate that the gene is cotranscribed with others in an operon. Download Table S1, XLSX file, 0.03 MB. Copyright © 2021 Dmitriev et al. 2021 Dmitriev et al. https://creativecommons.org/licenses/by/4.0/ This content is distributed under the terms of the Creative Commons Attribution 4.0 International license.

We tested whether the apparent expansion of the Rex regulon in S. aureus contributes to NO· resistance. We noticed that S. simiae encodes almost as many Rex-regulated genes as S. aureus, including *ldh*1, a gene not found in S. epidermidis or other CoNS, and one that is known to contribute to NO· resistance (11). We therefore compared the growth of S. aureus, S. simiae, and S. epidermidis while enduring NO· stress. Following the addition of NO·, S. aureus and S. simiae did not exhibit a growth defect, while S. epidermidis lagged in growth until the high concentration of NO· dissipated after 5 h of exposure (Fig. 1B). Since Rex is a repressor, we hypothesized that overexpressing it might prevent the production of dehydrogenases that are important for maintaining redox balance in the absence of respiration. Indeed, overexpression of Rex from the constitutive *lgt* promoter prevented growth of S. aureus in the presence of NO· but did not affect untreated cells (Fig. 1C). Taken together, these data suggest that the apparent expansion of the Rex regulon is necessary and sufficient for NO· resistance. Additionally, overexpression of Rex inhibited anaerobic growth, suggesting that any time respiration is hindered, derepression of the Rex regulon is essential for growth (Fig. 1D). Furthermore, it appears that this expansion occurred sometime after the last common ancestor shared by S. aureus and S. simiae diverged from the S. epidermidis lineage (Fig. S1) since both species are NO· resistant, while S. epidermidis is not.

10.1128/mBio.02188-21.1 FIG S1 Phylogenetic relationship of closely related staphylococcal species. Phylogenetic tree was generated using Geneious software (neighbor-joining method) from aligning 16S rRNA from each species. Points where the Rex regulon expanded (*rex*) or when the *srrAB* promoter became NO· responsive (srrAB*) are indicated in red. Download FIG S1, JPG file, 0.1 MB. Copyright © 2021 Dmitriev et al. 2021 Dmitriev et al. https://creativecommons.org/licenses/by/4.0/ This content is distributed under the terms of the Creative Commons Attribution 4.0 International license.

### SrrAB expression is responsive to NO· exposures in S. aureus only.

**FIG 2:**
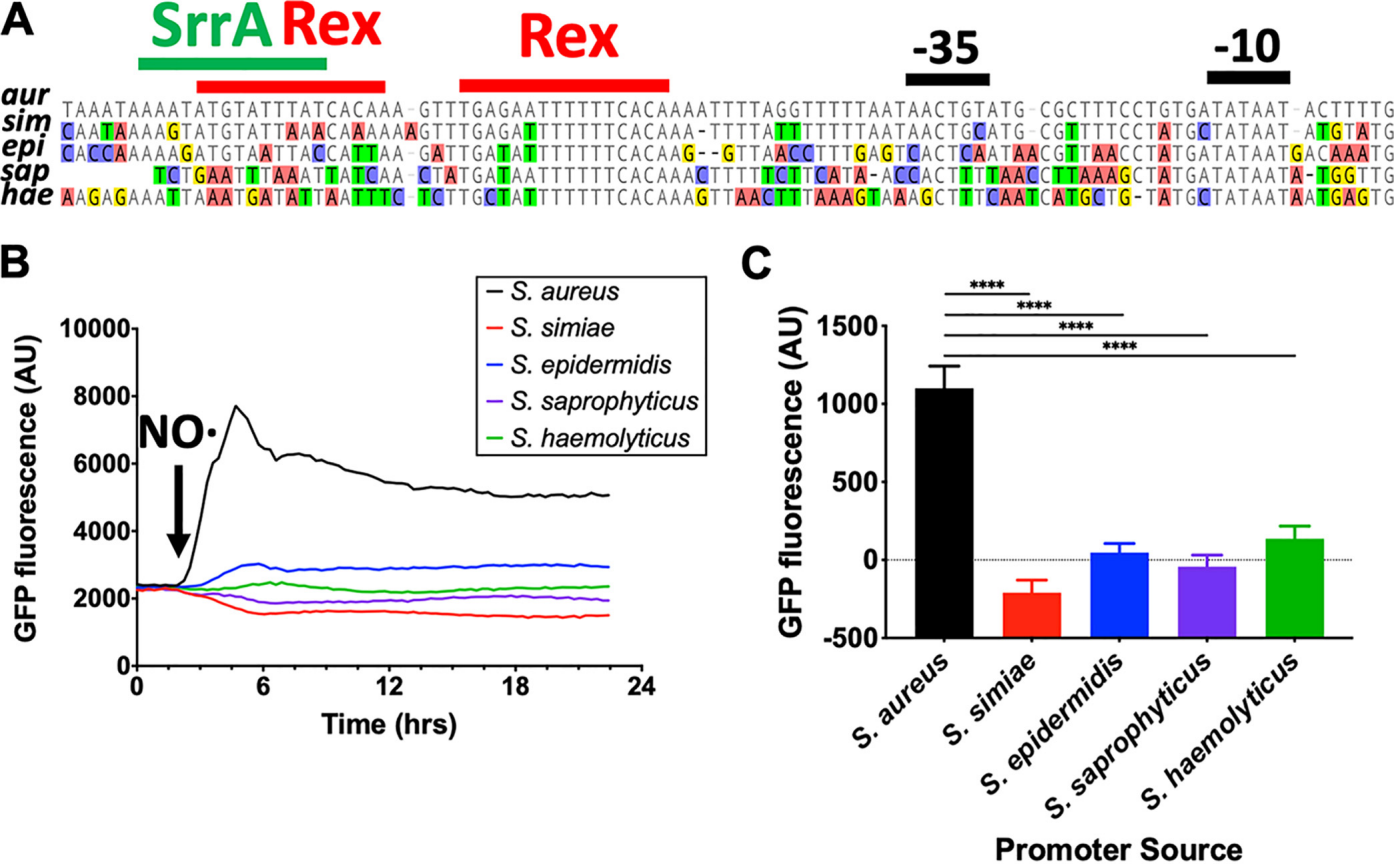
S. aureus *srrAB* alone responds to exogenous NO·. (A) Alignment of *srrAB* promoter regions from closely related staphylococcal species. Putative Rex (TGTGAW_6_TCACA) and SrrA (AAATAN_4_TTTAT) binding sights are outlined in red and green, respectively. (B) Expression of GFP driven by the S. aureus *srrAB* promoter responds to NO· (10 mM DETA-NO administered at OD_660_ of 0.2), whereas *srrAB* promoters from other species do not. (C) Quantification of p*_srrAB_*-GFP induction 30 min following NO· challenge from closely related staphylococcal species. Data were analyzed via one-way analysis of variance (ANOVA) with Dunnett’s correction for multiple comparisons (****, *P* ≤ 0.0001).

NO· exposure is known to induce the expression of SrrAB, which, in turn, drives expression of the SrrA regulon. Rex and SrrA both bind directly to the *srrAB* promoter, so we hypothesized that Rex and/or SrrA are responsible for the NO· responsiveness of S. aureus *srrAB* (9, 20). Since the putative binding sites for Rex and SrrA are not well conserved in CoNS (Fig. 2A), we tested whether *srrAB* promoters from any other species responded to the presence of NO·. Cloning the promoters for *srrAB* from S. aureus, S. simiae, S. epidermidis, S. saprophyticus, and S. haemolyticus so that each drove green fluorescent protein (GFP) expression showed that only the S. aureus *srrAB* promoter is NO· responsive (Fig. 2B and C). This did not correlate with basal SrrAB expression levels in the absence of NO· exposure (Fig. S2).

10.1128/mBio.02188-21.2 FIG S2 Basal expression of GFP driven by the indicated species *srrAB* promoter. Values were derived from the same time points as in Fig. 2C but in cultures where they were not treated with NO·. Download FIG S2, JPG file, 0.1 MB. Copyright © 2021 Dmitriev et al. 2021 Dmitriev et al. https://creativecommons.org/licenses/by/4.0/ This content is distributed under the terms of the Creative Commons Attribution 4.0 International license.

**FIG 3:**
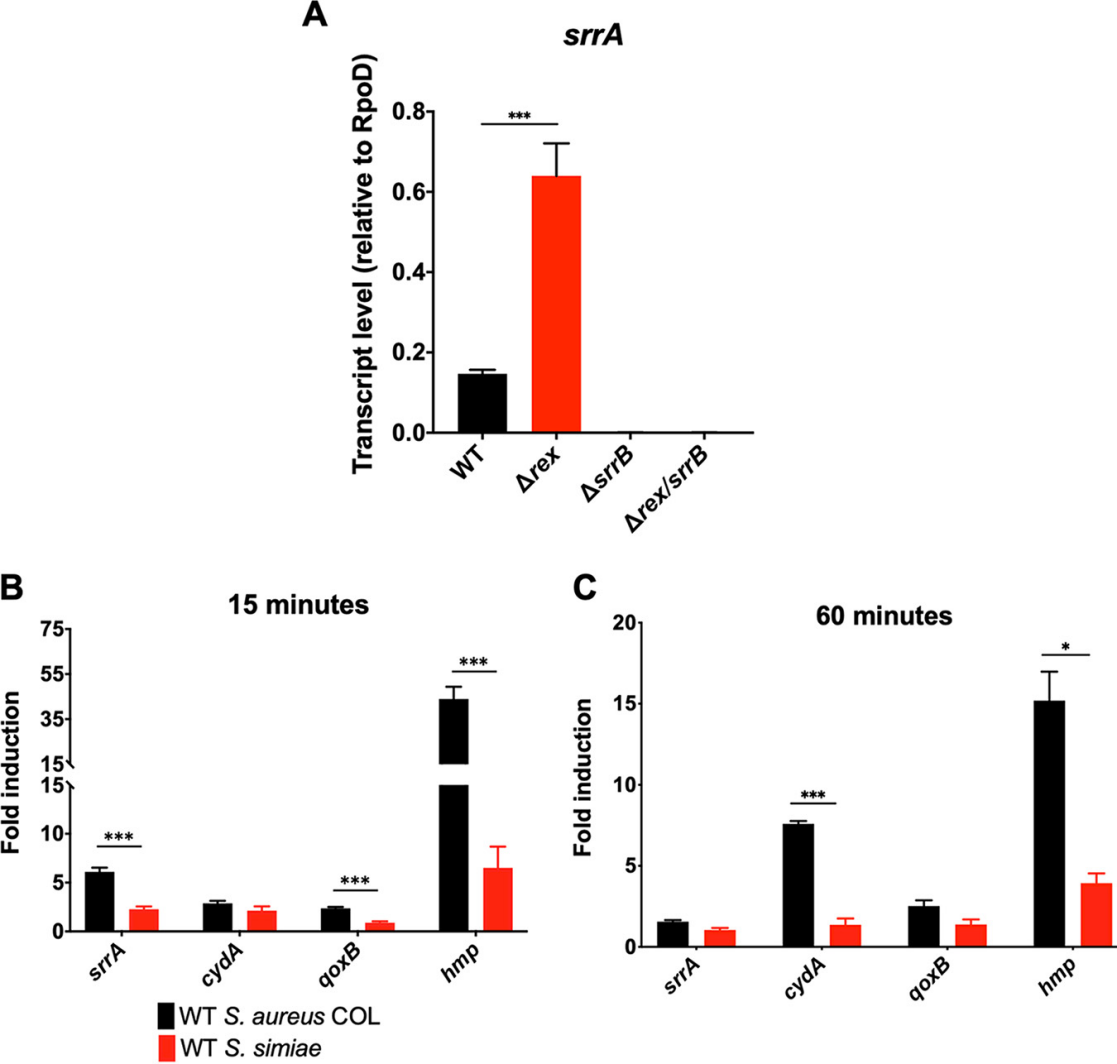
qRT-PCR analyses of the induction of *srrAB* and SrrAB-regulated genes in S. aureus and S. simiae upon exposure to exogenous NO·. (A) *srrA* transcript level analyzed via qRT-PCR and normalized to that of *rpoD* in wild-type (WT) S. aureus and indicated isogenic mutants in the absence of exogenous NO·; *n* = 3. Statistical significance was established via a one-way ANOVA with Dunnett’s posttest (***, *P* ≤ 0.0001). (B and C) Fold induction of indicated genes 15 min (B) or 60 min (C) following NO· exposures (administered as 10 mM DETA-NO; *n* = 3) relative to untreated expression levels. Expression levels were normalized to that of *rpoD*, and induction levels were compared between species for a given gene/time point using Student's *t* test using the Holm-Sidak method (***, *P* ≤ 0.0001; **, *P* ≤ 0.01; *, *P* ≤ 0.05).

The putative Rex binding sites are ∼20 bp upstream of the −35 sequence, which is not consistent with preventing RNA polymerase from accessing the *srrAB* promoter (Fig. 2A). However, deletion of *rex* resulted in a modest 5-fold induction of *srrAB* even in the absence of NO· (Fig. 3A). This, in turn, led to elevated levels of SrrA-activated cytochrome expression in some instances as well (Fig. S3A and B). Furthermore, the Δ*rex* mutant had no effect on *srrAB* expression in the presence of NO· (Fig. S3B). These observations are consistent with Rex-mediated repression of SrrAB expression as the source of NO· responsiveness in S. aureus. However, the Δ*srrB* mutant demonstrated virtually no expression of SrrAB and exhibited severe reduction in the expression of SrrAB-regulated genes both in the presence and the absence of NO· (Fig. 3A; Fig. S3A and B). Moreover, the double Δ*rex* Δ*srrB* mutant phenocopied the Δ*srrB* mutant (Fig. 3A; Fig. S3A and B). The epistatic relationship between Rex and SrrB on SrrAB expression is more consistent with Rex preventing the auto-induction of SrrAB expression by SrrA. Since the known Rex-repressed *ldh*1 was NO· inducible in S. simiae (Fig. S3C), the lack of induction of *srrAB* by NO· in S. simiae cannot be due to a defect in Rex derepression. Rather, the SrrA binding site is significantly divergent between S. aureus and CoNS, explaining the unique NO· responsiveness of SrrAB expression in S. aureus (Fig. 2A).

10.1128/mBio.02188-21.3 FIG S3 Raw transcript levels of indicated genes in WT or mutant backgrounds normalized to *rpoD* mRNA. (A) Expression levels of various SrrA-regulated genes in unstimulated cells. (B) Expression levels of various SrrA-regulated genes from NO·-stimulated cells. RNA was isolated from indicated strains 15 minutes after addition of NO· (10 mM DETA-NO) administered to aerobically cultured cells at OD_660_ of 0.5. (C) Induction of *ldh*1 transcript in both S. aureus and S. simiae upon exposure to NO· (10 mM DETA-NO for 15 min). Statistical significance was determined via Student’s *t* test (#, *P* ≤ 0.05; *, *P* ≤ 0.01; **, *P* ≤ 0.005; ***, *P* ≤ 0.001; ****, *P* ≤ 0.0001). Download FIG S3, JPG file, 0.2 MB. Copyright © 2021 Dmitriev et al. 2021 Dmitriev et al. https://creativecommons.org/licenses/by/4.0/ This content is distributed under the terms of the Creative Commons Attribution 4.0 International license.

### Elevated SrrAB activity in S. aureus allows for optimum respiratory capacity during NO· stress.

Since the SrrA regulon includes genes involved in cellular respiration and NO· detoxification, we reasoned that these genes may be expressed to a higher degree in S. aureus than S. simiae upon stimulation with NO·. As expected, *srrA*, *qoxB*, and *hmp* transcripts were more abundant in S. aureus than in S. simiae 15 min after NO· treatment (6-fold, 2-fold, and 43-fold, respectively) (Fig. 3B and Fig. S4A). Furthermore, 60 min after NO· exposure, *cydA* and *hmp* transcripts were more abundant in S. aureus by 8-fold and 15-fold, respectively (Fig. 3C and Fig. S4B). Therefore, since SrrAB is NO· responsive in S. aureus alone, this species overproduces downstream effectors such as cytochrome production and NO·-detoxifying enzymes compared to closely related S. simiae.

10.1128/mBio.02188-21.4 FIG S4 Raw transcript levels of indicated genes normalized to *rpoD* mRNA. (A) Genes normalized either without NO· addition or 15 minutes after NO· exposure (10 mM DETA-NO) administered to aerobically cultured cells at OD_660_ of 0.5. (B) Genes normalized either without NO· addition or 60 minutes after NO· exposure (10 mM DETA-NO) administered to aerobically cultured cells at OD_660_ of 0.5. All values represent the average of 3 independent biological experiments. Asterisk indicates S. aureus exhibiting significantly higher transcript levels than S. simiae (*P* ≤ 0.05, *n* = 3, Student’s *t* test). Download FIG S4, JPG file, 0.2 MB. Copyright © 2021 Dmitriev et al. 2021 Dmitriev et al. https://creativecommons.org/licenses/by/4.0/ This content is distributed under the terms of the Creative Commons Attribution 4.0 International license.

**FIG 4:**
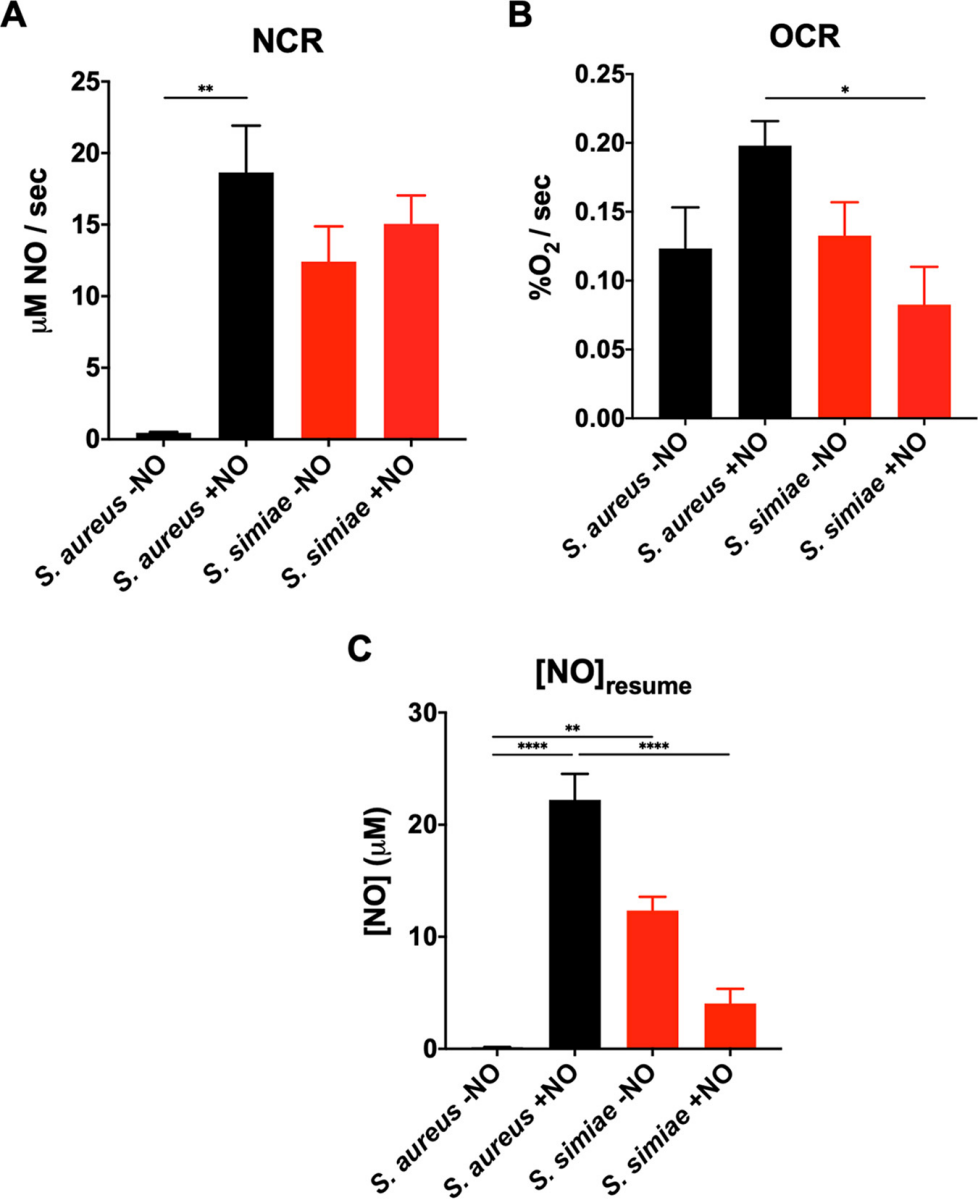
S. aureus alone exhibits elevated respiratory capacity and NO· detoxification upon exposure to exogenous NO·. (A) NO· consumption rate by cells either prestimulated with NO· (10 mM DETA-NO for 1 h) or unstimulated. (B) Oxygen consumption rate before or after NO· exposure (10 mM DETA-NO for 1 h) using a Clark-type electrode. Oxygen consumption was initiated by addition of 0.01% glucose to washed and resuspended cells (OD_660_, 1.0) (C) Concentration of NO· remaining in suspension when cells resume respiration. Cells were either prestimulated with NO· (10 mM DETA/NO for 1 h) or unstimulated. Statistical comparisons were carried out using a one-way ANOVA with Tukey’s posttest (****, *P* ≤ 0.0001; **, *P* ≤ 0.01; *, *P* ≤ 0.05).

A consequence of a relatively overactive SrrAB regulon is the optimization of respiratory activity in the presence of NO·. NO· will temporarily halt respiration through competitive binding of cytochrome heme cofactors. Once NO· levels have been reduced via enzymatic detoxification, however, cellular respiration can resume. We measured this *in vitro* by using amperometric probes to measure oxygen and NO· concentrations in cell suspensions of S. aureus COL and S. simiae in real time. Representative traces show both the spike and clearance of NO· and the halt and resumption of oxygen consumption via respiration (Fig. S5). Since Hmp is the primary means of NO· detoxification in these species and since it is induced much more in S. aureus due to overexpression of SrrAB, the NO· consumption rate was significantly higher in S. aureus than S. simiae upon stimulation (Fig. 4A). Interestingly, while it is known that S. aureus exhibits little or no·consumption without stimulation, S. simiae seems to express Hmp constitutively, as the NO· consumption rate was not affected by prior exposure to this immune radical (Fig. 4A). Similarly, since both QoxABCD and CydAB were induced by NO· more robustly in S. aureus, this species exhibited NO·-enhanced respiratory capacity, while S. simiae did not (Fig. 4B). Given that NO·-exposed S. aureus exhibits enhanced NO· detoxification and expresses relatively higher levels of cytochromes upon NO· exposure than S. simiae, we tested whether S. aureus could resume respiration in the presence of higher levels of NO· than its closely related species. Indeed, we found that S. aureus is able to resume respiration at extracellular NO· concentrations more than five times that of S. simiae (Fig. 4C), a trait likely to serve the pathogen at sites of inflammation.

10.1128/mBio.02188-21.5 FIG S5 Representative NO· and O_2_ consumption traces. Washed and suspended cells (OD_660_ of 1.0) were stimulated to begin oxygen consumption by the addition of 0.01% glucose. NO· (red line) was administered (100 μM PROLI-NO; half-life [*t*_1/2_], 1.8 s) to temporarily inhibit respiration, e.g., oxygen consumption (black line). Once the NO· was sufficiently consumed, respiration resumed until the oxygen was exhausted. Download FIG S5, JPG file, 0.3 MB. Copyright © 2021 Dmitriev et al. 2021 Dmitriev et al. https://creativecommons.org/licenses/by/4.0/ This content is distributed under the terms of the Creative Commons Attribution 4.0 International license.

## DISCUSSION

Compared to most coagulase-negative staphylococci, S. aureus is able to grow much better in the absence of respiration, whether being cultivated anaerobically or in the presence of respiratory inhibitors such as NO· (21). Here, we demonstrate that the expanded Rex regulon is necessary and sufficient for this trait as follows. In the absence of respiration, overexpressing the Rex repressor prevents derepression of the regulon. Consequently, these strains cannot grow anaerobically or in the presence of NO· (Fig. 1C and D). In addition, S. simiae, which also possesses an expanded Rex regulon, is also highly resistant to NO· compared to other coagulase-negative staphylococci (Fig. 1B). Various dehydrogenases and metabolite transporters comprise the Rex regulon, and while the substrates for these enzymes/transporters are largely unknown, they are predicted to be small organic acids and/or amino acids. The expanded Rex regulon would solve a problem with the metabolic strategy of S. aureus during NO· stress as we know it today: homolactic fermentation would not allow for incorporation of carbon into biomass. Indeed, host immune cells employ homolactic fermentation and convert one mole of glucose to two moles of lactate, resulting in redox-balanced energy production, but these cells are not replicating. For S. aureus to divide and generate a gram of biomass, it consumes 12 g of glucose, 11 for energy and 1 for biomass (21). If all the glucose is converted to lactate, all carbon would be excreted as waste. Rather, the ability of S. aureus to reduce exogenous substrates to regenerate NAD^+^ allows the organism to use some of the glucose carbon for the production of biomass. S. simiae may have evolved to use this metabolic strategy to thrive in the anaerobic primate gut, while S. aureus adopted it to thrive at sites of inflammation. Both environments would require efficient respiration-independent growth.

While the last common ancestor shared by S. aureus and S. simiae may have evolved an expanded Rex regulon to thrive anaerobically, the fact that S. aureus adapted to inflammatory radicals would require additional evolutionary changes. One change is the autoregulatory feedback loop of SrrAB (Fig. 3A). Rex prevents the auto-induction of *srrAB*, but when the Rex regulon is derepressed, SrrA maximizes *srrAB* transcription. Higher levels of phosphorylated SrrA leads to higher levels of cytochromes and NO·-detoxifying flavohemoprotein (Hmp) (Fig. 3B and C). This would allow S. aureus to “outcompete” host immune radical production and continue respiring despite their presence. Indeed, when exposed to NO·, S. aureus resumed respiration and oxygen consumption at NO· levels ≥5-fold higher than S. simiae (Fig. 4C). When S. simiae senses a buildup of NADH, it is most likely due to it entering the anaerobic environment of the primate gut. Therefore, it would not be necessary to induce cytochromes or Hmp. In contrast, a common reason for S. aureus to sense high NADH is because of host immune radicals, which inhibit respiration. In response, overproducing cytochromes, NO· detoxification, and Fe-S cluster repair systems provide a metabolic advantage aimed at overcoming the respiratory hinderances of host inflammation. This may be especially true in tissues where glucose is less abundant since respiration is key for metabolizing gluconeogenic substrates in S. aureus (22, 23).

Both Rex and SrrA have been shown to directly bind the *srrAB* promoter, and there are two potential Rex binding sites upstream of the −35 and one for SrrA (Fig. 2A) (9, 20). However, only one Rex site is active since there was only one shift when incubating recombinant Rex with the *srrAB* promoter (9). While we do not know definitively which site is bound, either could potentially interfere with SrrA auto-activation. One overlaps entirely with the predicted SrrA binding site, and the other is downstream where binding by Rex could interfere with the SrrA-RNA polymerase interactions. Furthermore, neither Rex binding sites are completely conserved among coagulase-negative staphylococci, including S. simiae. Moreover, the SrrA binding site is completely degenerate in all species other than members of the SAC (Fig. 2A). This implies that the SrrA auto-activation and the Rex repression of this operon evolved relatively recently in S. aureus. The SrrA requirement for the *srrAB* promoter likely stems from mutations that accumulated in the −35 region. Indeed, while the −10 is completely conserved, the −35 is highly variable, which is consistent with the requirement of SrrA for *srrAB* transcription in S. aureus, but with relatively constitutive expression in other species.

Another indicator that S. simiae has evolved to hypoxic or anaerobic environments is the constitutive NO·-consuming activity exhibited by this species. While the clonal complex 30 (CC30) lineage of S. aureus encodes both a NO· reductase and Hmp, most clones only harbor the gene for the flavohemoprotein (*hmp*). Similarly, S. simiae only encodes an Hmp for NO· detoxification. In S. aureus, Hmp is relatively scarce until the cell encounters NO· stress (Fig. 4A). In contrast, in S. simiae, Hmp is constitutively expressed and is not induced by exogenous NO· in the environment. It is known that Hmp expression in the absence of NO· can lead to ROS production, and therefore, the enzyme could be toxic in the presence of oxygen (24). The fact that *hmp* is constitutively expressed in S. simiae could indicate this organism is generally found in low-oxygen environments. Alternatively, like S. aureus, S. simiae also encodes an NO· synthase. Low-level NO· production by this nitrous oxide system (NOS) might be enough to prevent Hmp from spontaneously reducing molecular oxygen.

In the end, here, we present evidence that the expanded Rex regulon in certain species of staphylococci is necessary and sufficient for NO· resistance. We also suggest that this expansion originally served as an adaptation to low-oxygen environments but was co-opted by S. aureus to thrive at sites of inflammation. This required additional evolutionary adaptations, namely, the Rex-repressed and -autoregulated SrrAB system, which controls cytochrome production and NO· detoxification. This adaptation likely allows S. aureus specifically to avoid the cytotoxic effects of host NO·.

## MATERIALS AND METHODS

### Bacterial strains and growth conditions.

Strains used in this study are described in Table 1.All strains were grown in either brain heart infusion medium (BHI; Difco, Sparks, MD) or chemically defined PN medium supplemented with 0.5% glucose (25). Cultures were shaken at 250 rpm unless otherwise specified. Antibiotic selection in S. aureus (E. coli) was performed using the following concentrations: 25 μg·ml^−1^ kanamycin, 5 μg·ml^−1^ erythromycin, 20 μg·ml^−1^ chloramphenicol, and 100 μg·ml^−1^ ampicillin. All restriction enzymes were purchased from New England Biolabs (Ipswich, MA).

**TABLE 1:** Strains and plasmids used in this study

Strain or plasmid	Genotype	Source or reference
Strains		
S. aureus COL	Methicillin-resistant clinical isolate; laboratory strain	Laboratory strain
S. aureus LAC	Methicillin-resistant clinical isolate; laboratory strain	Laboratory strain
S. simiae	CCM 7213	Laboratory strain
S. epidermidis	RP62A	Laboratory strain
S. saprophyticus	ATCC 15305	Laboratory strain
S. haemolyticus	JCSC1435	Laboratory strain
AR1593	COL + pAD02	This study
AR1612	COL + pEP06	This study
AR1606	COL + pEP05	This study
AR1600	COL + pEP04	This study
AR1569	COL + pAD01	This study
AR0352	COL Δrex::Kn^r^	27
AR1626	COL ΔsrrB::Er^r^ (Φ11 NE588)	This study
AR1630	COL Δrex::Kn^r^, ΔsrrB::Er^r^ (Φ11 NE588)	This study
AR1315	COL + pOS1-P_lgt_	This study
AR1408	COL + pNV55	This study
NE588	SAUSA300_1441::Tn	28
Plasmids		
pBT2ts	E. coli/S. aureus shuttle vector	32
pBTK	1.4 kb *aph*-A3 allele cloned into SmaI of pBT2ts	29
pJF119	CAT allele (Cm^r^) replacement of ApaI/XhoI Er^r^ region of promoterless GFP fusion vector pCN52	27, 30
pAD01	*P_srrAB (_*_S. haemolyticus_*_)_* cloned into BamHI/EcoRI of pJF119	This study
pAD02	*P_srrAB (_*_S. aureus_*_)_* cloned into BamHI/EcoRI of pJF119	This study
pEP04	*P_srrAB (_*_S. saprophyticus_*_)_* cloned into BamHI/EcoRI of pJF119	This study
pEP05	*P_srrAB (_*_S. epidermidis_*_)_* cloned into BamHI/EcoRI of pJF119	This study
pEP06	*P_srrAB (_*_S. simiae_*_)_* cloned into BamHI/EcoRI of pJF119	This study
pOS1-P*_lgt_*	S. aureus complementation vector driven by the *lgt* promoter	31
pNV55	*rex* allele cloned into NdeI of pOS1-P*_lgt_*	This study

AR0352 was generated via allelic replacement using the E. coli-S. aureus shuttle vector pBTK as previously described (Cooke, PLoS One). AR1626 and AR1630 were created via Φ11 phage transduction of NE588 into S. aureus COL or AR0352, respectively. GFP reporter strains driven by *srrAB* promoters were constructed as follows. Homologous *srrAB* promoters were PCR amplified from S. aureus COL, S. simiae, S. epidermidis, S. haemolyticus, and S. saprophyticus genomic DNA, resulting in amplicons with 5′ BamHI and EcoRI restriction sites for directional ligation into the GFP reporter transcriptional fusion vector pJF119. Plasmids were then propagated through E. coli via electroporation (with ampicillin selection), harvested using a QIAprep Spin miniprep kit (Qiagen, Hilden, Germany), and then transformed into S. aureus restriction-deficient strain RN4220 (with chloramphenicol selection) (26). Plasmids were finally transduced into S. aureus COL using Φ11 phage lysates made from the transformed RN4220 strains.

### Rex regulon definition.

Genomes from S. aureus (COL; GenBank accession no. CP000046), S. simiae (CCM_7213; GenBank accession no. AEUN01000002), S. epidermidis (RP62A; GenBank accession no. CP000029), S. haemolyticus (JCSC1435; GenBank accession no. NC_007168), S. saprophyticus (ATCC 15305; GenBank accession no. AP008934), S. pseudintermedius (HKU10-03; GenBank accession no. NC_014925), S. lugdunensis (HKU09-01; GenBank accession no. NC_013893), S. warneri (SG1; GenBank accession no. CP003668), S. pasteuri (SP1; GenBank accession no. NC_022737), and M. caseolyticus (JCSC5402; GenBank accession no. NC_011999) were queried using Geneious Prime v2021.1.1 for Rex consensus sites (TTGTGAW_6_TCACAA) within 400 bp of a start codon and with ≤2 mismatches.

### Growth curves.

Cells were grown at 37°C in 200 μl PN medium and shaken aerobically (1 mm orbital) on a Synergy HTX plate reader (Biotek, Winooski, VT) or in an anaerobic chamber (Coy, Grass Lake, MI). Optical density at 660 nm (OD_660_) measurements were taken every 15 min for 24 h. The medium was supplemented with chloramphenicol for antibiotic selection when appropriate. We added 10 mM NOC-12 (EMD Millipore Sigma, Temecula, CA) and 1 mM diethylamine NONOate (DEA-NO) (Sigma-Aldrich, St. Louis, MO) when cultures concurrently reached an OD_660_ of 0.15, and then growth was allowed to resume.

### GFP reporter experiments.

Cells were grown at 37°C in 200 μl BHI medium supplemented with chloramphenicol and shaken aerobically (1 mm orbital) on a Synergy HTX plate reader (Biotek, Winooski, VT) for 24 h. When cultures concurrently reached an OD_660_ of 0.2, DETA-NO (Acros Organics, Fair Lawn, NJ) was added to a final concentration of 10 mM, and then growth was allowed to resume.

### Quantitative reverse transcriptase real-time PCR.

### (i) RNA extraction.

Cells were grown at 37°C in 60 ml of BHI medium in 500-ml baffled flasks. At an OD_660_ of 0.5, a 25-ml sample of cells was collected and mixed with 25 ml of ice-cold 1:1 ethanol/acetone in order to prevent RNA degradation before immediately being stored at −80°C until further use. After adjusting the remaining culture to a volume of 25 ml, DETA-NO was added to a final concentration of 10 mM, and cells were shaken for an additional 15 or 60 min under the same conditions. After 15 or 60 min, the 25-ml culture was collected and stored at −80°C in ethanol/acetone as previously described. Frozen cell suspensions were thawed at room temperature, pelleted via centrifugation, and resuspended in 250 μl of TE buffer, pH 8.0. They were then sequentially frozen in a dry ice/ethanol bath and thawed at 60°C a total of three times before being transferred to Lysing matrix B tubes (MP Biomedicals, Solon, OH). RNA extraction was further carried out with a PureLink RNA minikit (Invitrogen, Carlsbad, CA) per the manufacturer’s instructions with additional modifications. Briefly, tubes were bead beat for 60 s in a standard cell disruptor and then placed on ice for 5 min before the addition of 650 μl lysis buffer containing 10 μl β-mercaptoethanol and 1 ml buffer and completion of a second identical bead beating step. Following centrifugation and the standard binding and wash steps with optional on-column PureLink DNase treatment, RNA eluted in 50 μl of RNase-free water was further treated with 1 μl of off-column DNase I (New England BioLabs, Ipswich, MA) at 37°C for 60 min to ensure complete removal of contaminating DNA. Reaction mixtures were deactivated at 75°C for 10 min and mixed with both 350 μl lysis buffer and 250 μl 100% ethanol before being transferred to spin cartridges and eluted as instructed by the manufacturer.

### (ii) qRT-PCR.

RNA was quantified and assessed for purity via spectrophotometry. Quantitative reverse transcriptase real-time PCR (qRT-PCR) was performed using the Power SYBR green RNA-to-Ct 1-step kit (Applied Biosystems, Vilnius, Lithuania) as per the manufacturer’s instructions with 50 ng of RNA per reaction. Utilized primers are listed in Table 2, and primer efficiencies were determined empirically by creating a standard curve of amplification cycle (*C_T_*) values plotted against various concentrations of genomic DNA used for amplification. Primer efficiencies ranged from 1.76 to 2.02. For a given reaction, initial transcript abundance was determined for genes of interest in relation to *rpoD* housekeeping gene abundance by the following equation:

$$\frac{T_{\mathrm{GOI}}}{T_{\mathrm{rpoD}}}=\frac{E_{\mathrm{rpoD}}{}^{Ct_{\mathrm{rpoD}}}}{E_{\mathrm{GOI}}{}^{Ct_{\mathrm{rpoD}}}}$$

**Table 2:** Primers used in this study

Primer	Sequence	Use
srrAB-RT.2A	TGCCTGAAATGGATGGTATCC	qRT-PCR
srrAB-RT.2B	AACACGGTTTGTTTCTTCACC	qRT-PCR
cydA-RT.1A	CATTTCGATACATCTTCCCATGCC	qRT-PCR
cydA-RT.1B	ATCTGCTAAGAAACTCAATAGTCC	qRT-PCR
qoxB-RT.3A	GTTGTACTTGGCATGTTCGCC	qRT-PCR
qoxB-RT.3B	GGCATTATGGTGCATCTTACC	qRT-PCR
hmp-RT.1A	TGACTTTAGTGAATTTACACCAGG	qRT-PCR
hmp-RT.1B	CGTTTAACGCCAAAAGTTAAATGG	qRT-PCR
rpoD-RT.1A	AACTGAATCCAAGTGATCTTAGTG	qRT-PCR
rpoD-RT.1B	TCATCACCTTGTTCAATACGTTTG	qRT-PCR
srrA-Sim-RT.1A	GTAGATGATGAGGATAGAATC	qRT-PCR
srrA-Sim-RT.1B	ATGCAGGCATAATTATTTTCC	qRT-PCR
cydA-Sim-RT.1A	CATTTTGATACGTCTTCTCATGC	qRT-PCR
cydA-Sim-RT.1B	ATCAGCTAAGAAACTCAATACACC	qRT-PCR
qoxB-Sim-RT.3A	AATCTTTAACGCAAAAGGGCC	qRT-PCR
qoxB-Sim-RT.3B	TAGAAAAATGGCGAACATGCC	qRT-PCR
hmp-Sim-RT.3A	TAAAATGTTTAAGGCACATCC	qRT-PCR
hmp-Sim-RT.3B	TCAATATTAACTGCTGCAGCC	qRT-PCR
rpoD-Sim-RT.1A	TACGGATGAGAAACTAAATCC	qRT-PCR
rpoD-Sim-RT.1B	CCTTGTTCAATTCGTTTTGCC	qRT-PCR
COL-srrA.1A	gggggatccTGAAGGACGTGTATTGACGCC	Construction of pAD02
COL-srrA.1B	ggggaattcGACATACAGGTCATACCTCCC	Construction of pAD02
Sapro-srrA.1A	gggggatccGGTAGAGTACTTACGCCACAC	Construction of pEP04
Sapro-srrA.1B	ggggaattcTGACATACGTATATACCTCCC	Construction of pEP04
Haemo-srrA.1A	gggggatccTGAGGGCAGAGTACTGACACC	Construction of pAD01
Haemo-srrA.1B	ggggaattcTGTCATTTTGTTTATACCTCC	Construction of pAD01
RP62A-srrA.1A	gggggatccAAGGAAGAGTTCTTACACCAC	Construction of pEP05
RP62A-srrA.1B	ggggaattcGTCATACTTTCTACTACCTCC	Construction of pEP05
Sim-srrA.1A	gggggatccAGATAAGCGTGTGCTAACACC	Construction of pEP06
Sim-srrA.1B	ggggaattcGACATACAGGTTCTACCTCCC	Construction of pEP06

where $\frac{T_{\mathrm{GOI}}}{T_{\mathrm{rpoD}}}$ is the ratio of transcript abundance for any gene of interest to that of *rpoD*, *E* is the efficiency for the corresponding primer set, and *C_T_* is the amplification cycle at which the arbitrary threshold fluorescence was met. Fold induction was determined by dividing the calculated transcript ratio for a given gene expressed under NO· stress by its corresponding ratio for expression in the absence of NO·.

### Determination of nitric oxide and oxygen consumption.

Cells were grown in 200 ml of BHI in 2,000-ml flasks at 37°C and 200 rpm. At an OD_660_ of 0.5, cells were harvested and immediately spun down in 250 ml Sorvall centrifuge tubes. Alternatively, at an OD_660_ of 0.5, diethylene triamine NONOate (DETA-NO) was added to a final concentration of 10 mM, and cultures were shaken for an additional hour before being harvested in the same way. After being washed once with phosphate-buffered saline (PBS), cells were pelleted once more and resuspended to a final OD_660_ of 1.0 in PBS bubbled with air for 2 h. For a typical experiment, 60 ml of culture was transferred to a 100-ml beaker containing a magnetic stir bar, and all steps were conducted at 37°C. The culture was stirred at high intensity for 15 min to ensure maximal aeration before being sealed with a 3-holed rubber stopper, leaving no headspace in the beaker. ISO-NOP and ISO-OXY-2 amperometric probes (World Precision Instruments, Sarasota, FL) were inserted through the stopper along with an air-tight pipette tip to seal the injection port when not in use. The entire apparatus was air-tight, and both probes were allowed to polarize to a final minimal current level before conducting an experiment. With the culture being stirred at moderate intensity, glucose was added to a final concentration of 0.1% in order to initiate respiration. After allowing oxygen to be consumed for 2 min, as indicated by the probe tracing, the rapidly releasing NO· donor proline NONOate (PROLI-NO) (Cayman Chemical, Ann Arbor, MI) was added to a final concentration of 100 μM (resulting in an immediate release of 200 μM NO·). Continuous measurements were taken until all dissolved oxygen was consumed. NO· concentration was determined via comparison to a standard curve of PROLI-NO injections at doubling concentrations, while % O_2_ present was determined by setting the baseline current and the minimally detected current at the end of an experiment to 100% O_2_ and 0% O_2_, respectively.
