# Supplementary figures and images for: The Intersection of the Staphylococcus aureus Rex and SrrAB Regulons: an Example of Metabolic Evolution That Maximizes Resistance to Immune Radicals
Source: mBio. 2021 Nov 16;12(6):e02188-21. doi: 10.1128/mBio.02188-21 (PMC8593685; doi:10.1128/mBio.02188-21)

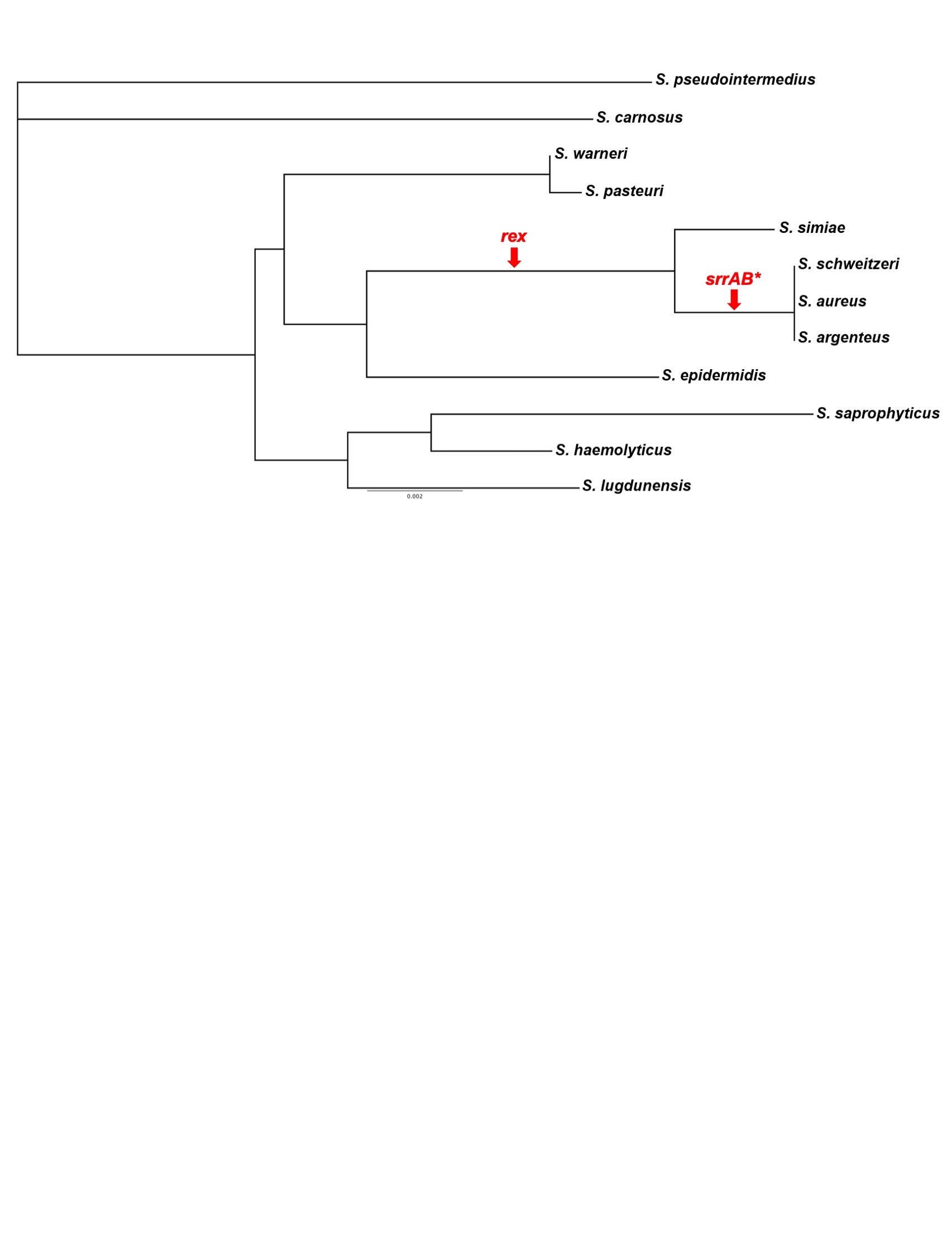

Supplement: FIG S1 [file mbio.02188-21-sf001.jpg]

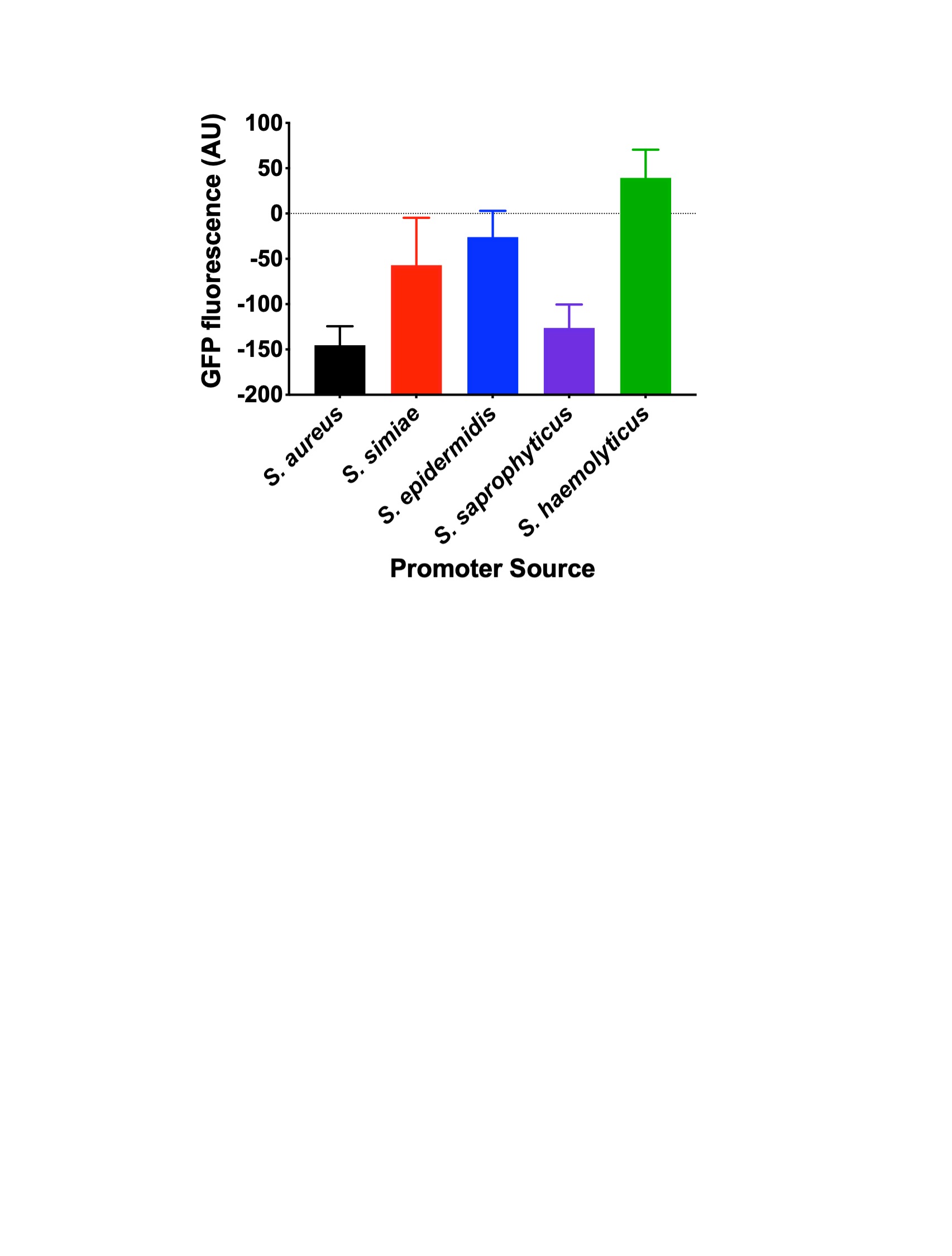

Supplement: FIG S2 [file mbio.02188-21-sf002.jpg]

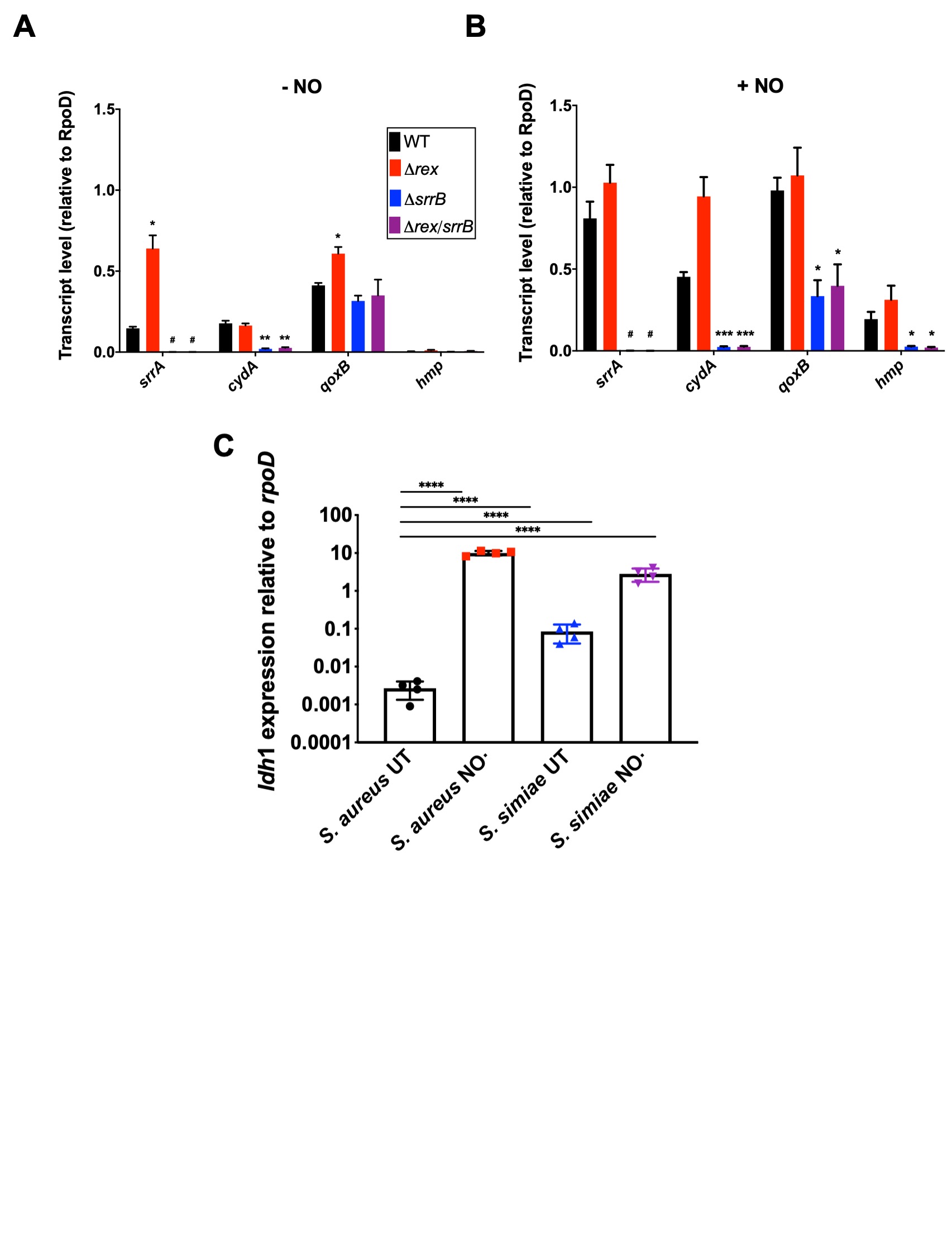

Supplement: FIG S3 [file mbio.02188-21-sf003.jpg]

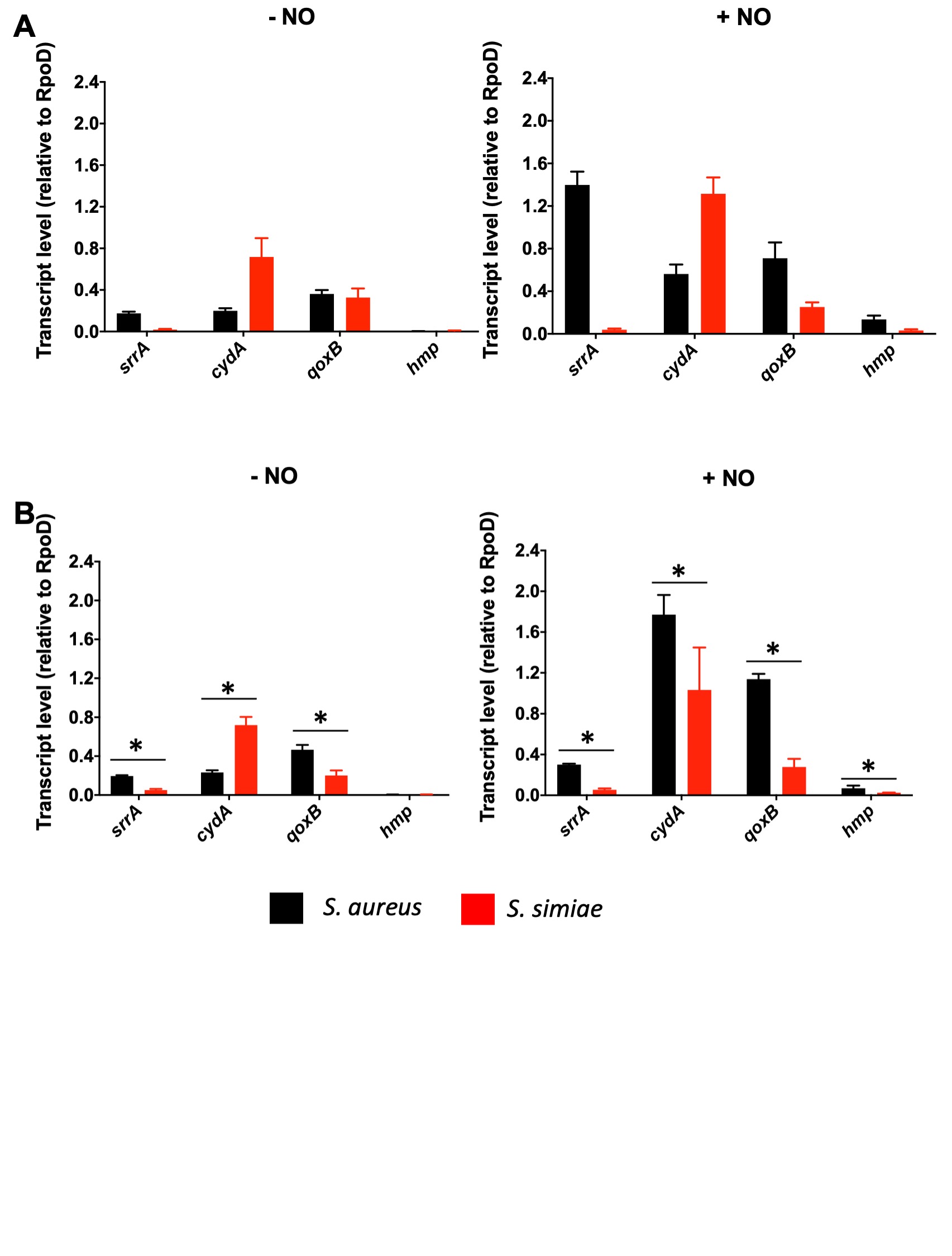

Supplement: FIG S4 [file mbio.02188-21-sf004.jpg]

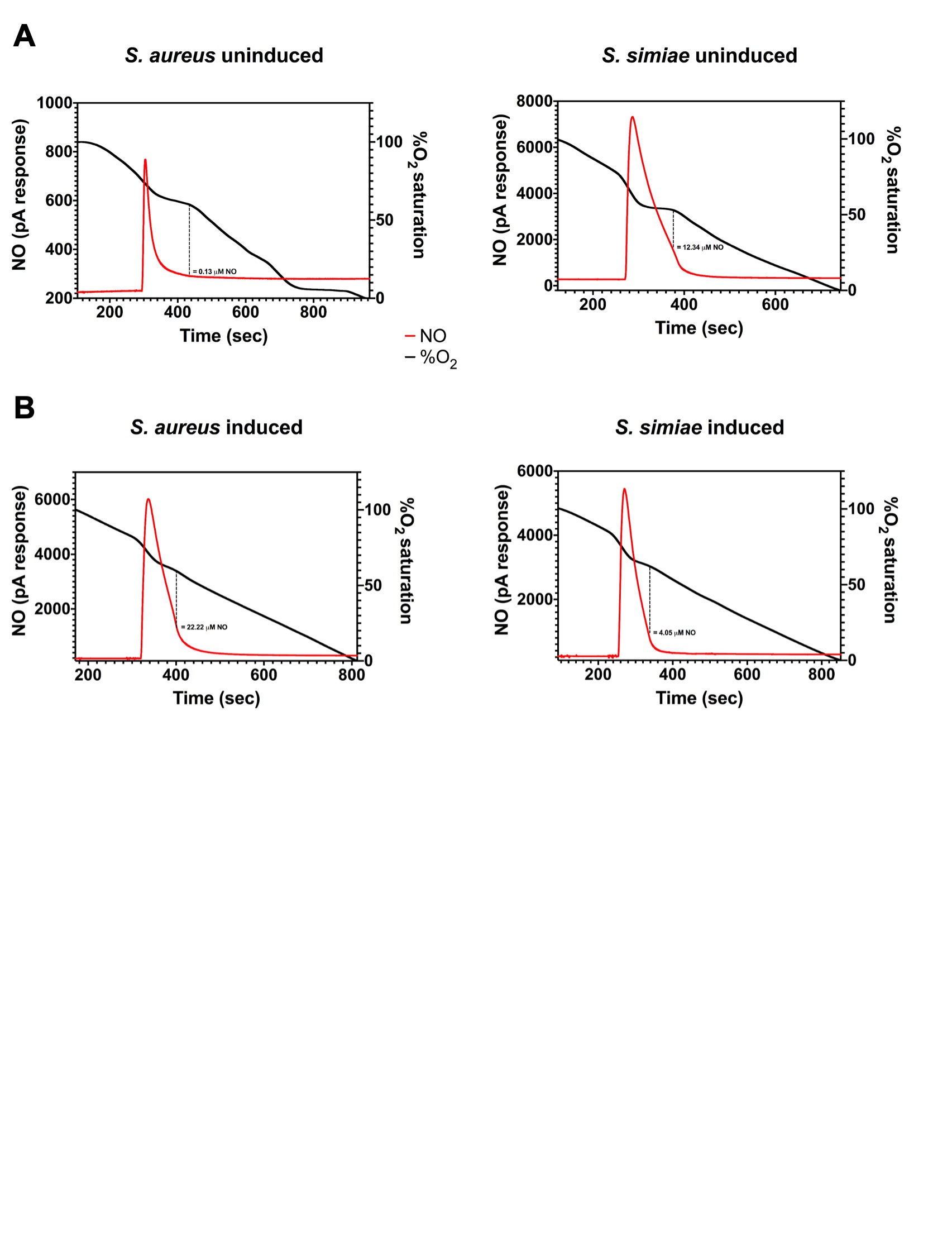

Supplement: FIG S5 [file mbio.02188-21-sf005.jpg]
